# Prosthesis selection for reconstruction of superior vena cava: comparison of midterm patency rates

**DOI:** 10.1093/icvts/ivae194

**Published:** 2024-11-26

**Authors:** Han Cho, Suk Ho Sohn, Jae Woong Choi, Ho Young Hwang, Kyung Hwan Kim, Kwon Joong Na, Chang Hyun Kang

**Affiliations:** Department of Thoracic and Cardiovascular Surgery, Seoul National University Hospital, Seoul National University College of Medicine, Seoul, Republic of Korea; Department of Thoracic and Cardiovascular Surgery, Seoul National University Hospital, Seoul National University College of Medicine, Seoul, Republic of Korea; Department of Thoracic and Cardiovascular Surgery, Seoul National University Hospital, Seoul National University College of Medicine, Seoul, Republic of Korea; Department of Thoracic and Cardiovascular Surgery, Seoul National University Hospital, Seoul National University College of Medicine, Seoul, Republic of Korea; Department of Thoracic and Cardiovascular Surgery, Seoul National University Hospital, Seoul National University College of Medicine, Seoul, Republic of Korea; Department of Thoracic and Cardiovascular Surgery, Seoul National University Hospital, Seoul National University College of Medicine, Seoul, Republic of Korea; Department of Thoracic and Cardiovascular Surgery, Seoul National University Hospital, Seoul National University College of Medicine, Seoul, Republic of Korea

**Keywords:** Superior vena cava, Vascular graft, Polytetrafluoroethylene, Bovine pericardium, Patency

## Abstract

**OBJECTIVES:**

This study compared the mid-term patency of expanded polytetrafluoroethylene grafts without rings versus that of bovine pericardial conduits used for superior vena cava reconstruction for various thoracic diseases.

**METHODS:**

Among 80 patients who underwent superior vena cava resection and reconstruction between 2009 and 2023 at our institution, 31 patients who received polytetrafluoroethylene grafts without rings (Polytetrafluoroethylene group) and 28 patients who received bovine pericardial conduits (Bovine group) were enrolled. Median follow-up durations were 19.5 and 64.6 months in the Polytetrafluoroethylene and Bovine groups, respectively. Primary outcome was midterm graft patency rate, and secondary outcomes were early and midterm clinical outcomes, including all-cause mortality and superior vena cava reintervention.

**RESULTS:**

Operative mortality was 1.7%. Cumulative incidence of all-cause mortality was not significantly different between the groups. Graft occlusion was detected in 22 patients. Cumulative incidence of graft occlusion was 24.2%, 36.4%, 42.4%, 48.5% and 60.6% at 1 month, 3 months, 6 months, 1 year and 2 years, respectively, in the Bovine group, whereas no graft occlusion was observed in the Polytetrafluoroethylene group (*P *=* *0.007). Although the incidence of graft occlusion was higher in the Bovine group, cumulative incidence of reintervention was not significantly different between the groups (0.0% vs 3.0% in Polytetrafluoroethylene vs Bovine groups at 1 year, *P *=* *0.406). Multivariate analysis demonstrated that bovine pericardial conduit (polytetrafluoroethylene graft as reference) and left brachiocephalic vein reconstruction (right brachiocephalic vein reconstruction as reference) were significant risk factors for graft occlusion.

**CONCLUSIONS:**

In superior vena cava reconstruction, polytetrafluoroethylene grafts without rings were superior to bovine pericardial conduits in terms of midterm graft patency.

## Graphical abstract

**Figure:**
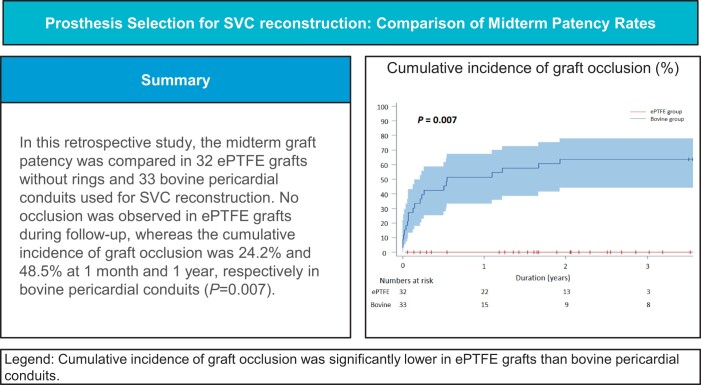

## INTRODUCTION

Advanced thymic epithelial tumours infiltrating the superior vena cava (SVC) can be surgically treated by resection and prosthetic reconstruction of the SVC via a multimodal therapeutic approach if it is radically resectable [1–4]. When performing the resection and reconstruction of the SVC, simple excision with primary or patch repair is possible for patients with limited involvement, whereas complex replacement of the SVC and 1 or both brachiocephalic veins (BCVs) is inevitable for patients with substantial vessel involvement [5, 6]. For prosthetic replacement of the SVC, various prosthetic materials and reconstruction strategies have been introduced [5]. However, the optimal prosthesis selection and best surgical strategy for SVC reconstruction in safety and efficacy are still limited.

At our institution, surgical management for advanced mediastinal tumours has been performed with satisfactory outcomes [7], and the strategies for SVC reconstruction have changed over time. Bovine pericardial conduits were mainly used in the earlier period, while expanded polytetrafluoroethylene (ePTFE) grafts without rings have been exclusively used in the later period. This study aimed to compare the midterm patency of ePTFE grafts to that of bovine pericardial conduits used for SVC reconstruction in various thoracic diseases.

## MATERIALS AND METHODS

The study protocol was reviewed by the Institutional Review Board and approved as a minimal risk retrospective study (approval date: 24 August 2023, approval number: H-2308-093-1458) that did not require individual consent based on the institutional guidelines for waiving consent.

### Patient enrolment

Patients who underwent SVC resection for various thoracic diseases between January 2009 and July 2023 at our institution were screened for study enrolment. Inclusion criteria were SVC reconstructions using ePTFE grafts without rings (ePTFE group) or bovine pericardial conduits (Bovine group). Exclusion criteria were primary repair, patch repair or SVC reconstructions using other materials, including autologous pericardium and ringed ePTFE grafts.

### Operative techniques

All procedures were performed under median sternotomy. The tumour was separated from its surrounding tissues for proper exposure, and the extent of the tumour was evaluated. If SVC invasion was suspected, whether to perform SVC repair or replacement after *en bloc* tumour excision was determined at the surgeons’ discretion. Proximal and distal control were obtained by dissection of the BCVs distal to the tumour and by dissection of the SVC proximal to the tumour, and they were encircled with vessel loops. The azygos vein was ligated and divided in most cases. Before the vessels were clamped, intravenous heparin was administered to prevent thrombus formation. After clamping, the SVC was resected *en bloc* and reconstructed with the selected prosthesis.

In earlier period, bovine pericardial conduits were mainly used because they were considered optimal substitutes due to their biocompatibility and technical ease [8, 9]. Various strategies were adopted according to the extent of tumour invasion, including ipsilateral reconstruction (from the right BCV or distal SVC to the proximal SVC) with/without left BCV resection and contralateral reconstruction [from the left BCV to the SVC or right atrial appendage (RAA)] with/without right BCV resection. In several patients with SVC and BCV invasion, bilateral vessel reconstruction was performed. Cardiopulmonary bypass was used in 13 patients (46.4%) in the Bovine group. The diameter of the grafts was determined by intraoperative measurements, and a bovine pericardial conduit was made following the methods introduced in a previous study [9]. The bovine pericardium was wrapped around a syringe to obtain the appropriate diameter and sutured longitudinally using a linear stapler.

**Figure 1:**
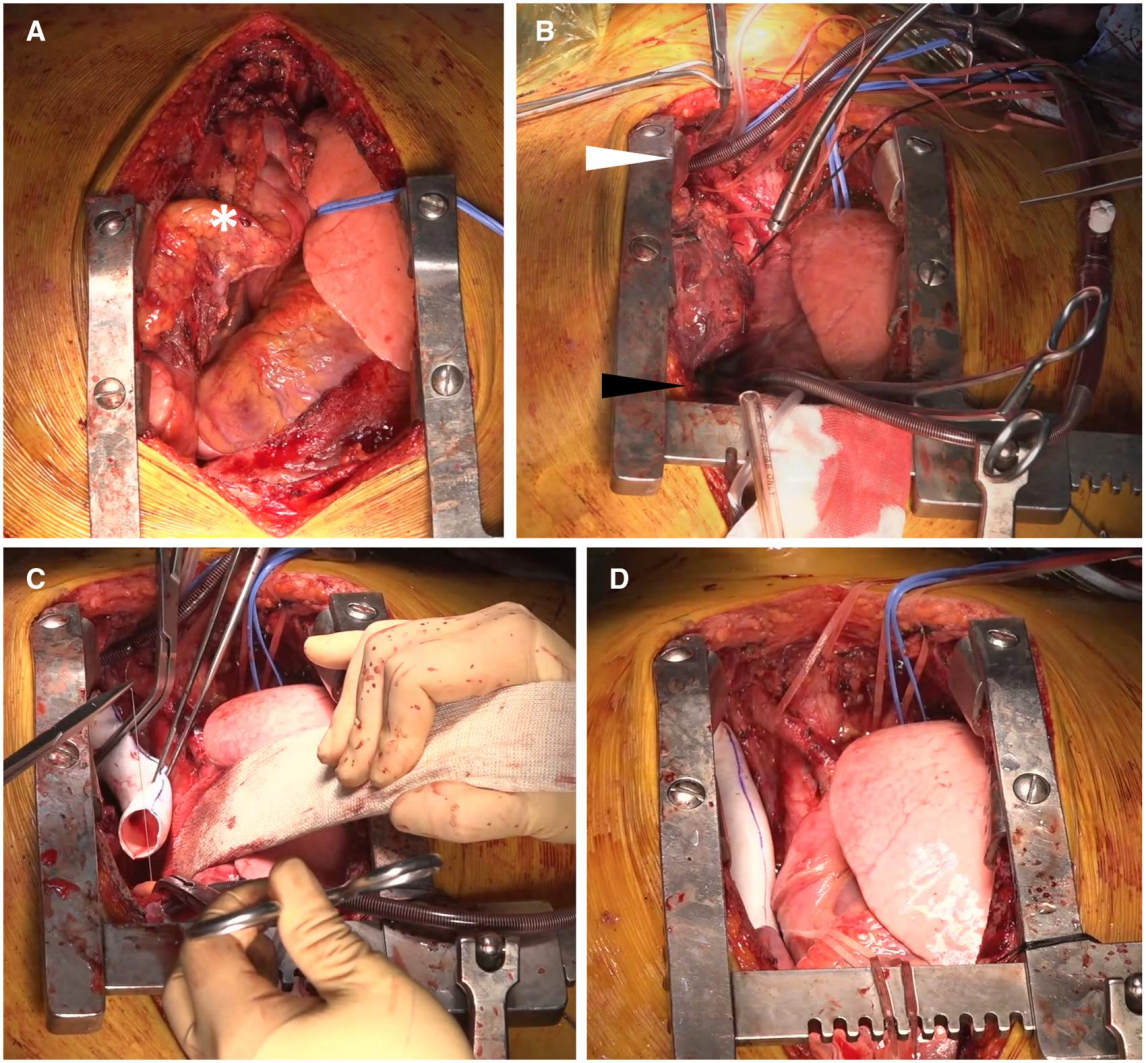
Operative techniques for SVC reconstruction using an ePTFE graft without rings. (**A**) The tumour (white asterisk) was separated from its surrounding tissues for proper exposure, and the extent of the tumour was evaluated. (**B**) An intraoperative venovenous shunt from the right brachiocephalic vein (white arrow) to the right atrial appendage (black arrow) was established using two 20 Fr cannulas and a connector. (**C**) After the SVC was clamped proximally and distally, *en bloc* resection of the SVC was performed. The ePTFE graft without rings was sutured in place with 5–0 PTFE running suture, starting with the distal anastomosis to the right BCV stump 1st, followed by the proximal anastomosis to the SVC stump. (**D**) After the anastomoses were complete, the cannulas were removed, and the heparin was reversed with protamine. BCV: brachiocephalic vein; ePTFE: expanded polytetrafluoroethylene; SVC: superior vena cava.

In the later period of use of ePTFE grafts without rings, an intraoperative venovenous shunt from one of the BCVs to the RAA was routinely used. We used an 18- or 20-Fr cannula according to the size of the BCV. After heparinization, a purse-string suture at the distal BCV was placed, and a cannula was inserted. Proximally, another purse-string suture was placed at the RAA, and another cannula was inserted. Then, the 2 cannulas were connected to form a venovenous shunt. After the SVC was clamped proximally and distally, *en bloc* resection of the SVC was performed, and the blood flow via the venovenous shunt was run passively by a pressure gradient from the BCV to the RAA without the aid of a pump. The graft was sutured in place with a 5–0 PTFE running suture, starting with distal anastomosis to the right BCV stump 1st, followed by proximal anastomosis to the SVC stump. Before the final suture was tied, the distal clamp was released to remove any air or thrombus in the graft. After the anastomoses were complete, the cannulas were removed and heparin was reversed with protamine (Fig. 1, Video 1).

### Anticoagulation

In the earlier period, low-molecular-weight heparin was given for anticoagulation in the absence of bleeding, and was converted to oral warfarin before discharge with a target international normalized ratio of 2.0–2.5. Patients were selectively transitioned to antiplatelet agents at the surgeon’s discretion. In the later period of use of ePTFE grafts, low-molecular-weight heparin was administered only 5–7 days after surgery and discontinued before discharge. Instead of warfarin, an antiplatelet agent (aspirin 100 mg daily) was administered beginning the day after surgery and continued at the outpatient follow-up.

### Evaluation of clinical outcomes

Operative mortality was defined as any death within 30 days after surgery or during the same hospital admission. After discharge, all patients underwent regular postoperative follow-up in the outpatient clinic at 3–6-month intervals. For the patients who were lost to follow-up, their survival or their dates of death were confirmed by the data from the National Health Insurance Service. SVC reintervention was defined as any intervention related to the occlusion or stenosis of the reconstructed SVC graft during follow-up. Clinical follow-up was terminated on 31 July 2023. The survival data were complete for 100.0% of the patients. The median follow-up durations were 19.5 months (interquartile range = 3.6, 31.8) and 64.6 months (interquartile range = 33.8, 105.2) in the ePTFE and Bovine groups, respectively.

### Evaluation of graft patency

Graft patency was evaluated via computed tomography (CT) using contrast media, which was performed mainly as a screening or surveillance tool for tumour recurrence. CT evaluation during follow-up was performed at 3-, 6- or 12-month intervals at the surgeons’ discretion considering the primary disease. Among the 59 patients, 41 patients underwent CT evaluation within 30 days after surgery, and the remaining 18 patients underwent CT evaluation within 1 year after surgery. Two radiologists blindly reviewed all CT evaluations and reached a consensus on their interpretations of graft patency.

### Statistical analysis

The statistical analysis was performed using SPSS statistics software version 27.0 (IBM Inc., Armonk, NY, USA) and SAS software version 9.4 (SAS Institute, Cary, NC, USA). Continuous variables are presented as the mean with standard deviation for normally distributed data or median with interquartile range for data that were not normally distributed, while categorical variables are presented as the number and percentage of subjects. Comparisons of baseline demographics, operative data and early clinical outcomes were performed using a chi-squared test or Fisher’s exact test for categorical variables and Student’s *t*-test or the Wilcoxon rank-sum test for continuous variables. Overall survival was estimated using the Kaplan–Meier method, and comparisons between the groups were performed using Cox proportional hazard regression with robust sandwich covariance matrix estimates. The cumulative incidences of graft occlusion and SVC reintervention were evaluated using the Fine-Gray subdistribution hazard model with penalized maximum likelihood, considering any death as a competing risk. Restricted mean survival time analysis was also performed for graft occlusion and SVC reintervention. To identify the risk factors for graft occlusion, univariate and multivariate analyses were performed using the Fine-Gray subdistribution hazard model. The variables in the preoperative risk factors and operative data were selected as covariates for the model. Variables with a *P* value <0.20 in the univariate analysis were included in the multivariate analysis with the backward method. All tests were two-tailed, and a *P* value <0.05 was considered to indicate statistical significance.

## RESULTS

### Patient characteristics

In 80 patients who were screened for enrolment, primary or patch repair was performed in 15 patients, and SVC reconstruction with a graft was performed in 65 patients. After excluding 3 patients with ringed ePTFE grafts and 3 patients with autologous pericardium, 59 patients were enrolled in this study. There were 31 patients who received an ePTFE graft without rings and 28 patients who received a bovine pericardial conduit. Bovine pericardial conduits were used before December 2019, and ePTFE grafts without rings were used beginning in January 2020.

**Figure 2:**
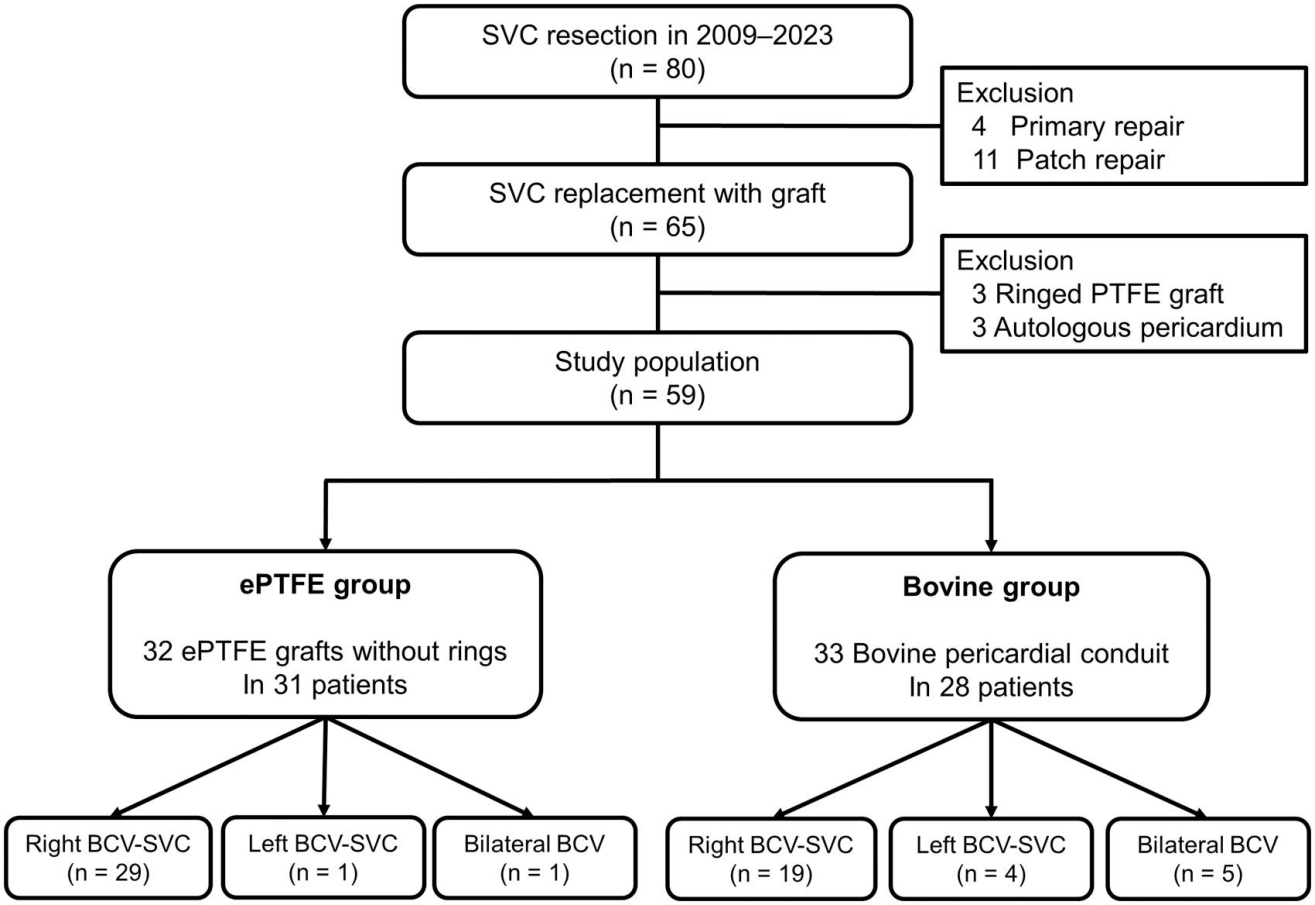
Flow diagram of the study population. BCV: brachiocephalic vein; ePTFE: expanded polytetrafluoroethylene; SVC: superior vena cava; PTFE: polytetrafluoroethylene.

In the ePTFE group, 32 reconstructions using ePTFE grafts were performed in 31 patients; 29 reconstructions involved right BCV-SVC reconstruction, 1 involved left BCV-SVC reconstruction and 1 involved bilateral BCV reconstruction. In the Bovine group, 33 reconstructions using bovine pericardial conduits were performed in 28 patients; 19 reconstructions involved right BCV-SVC reconstruction, 4 involved left BCV-SVC reconstruction and 5 involved bilateral BCV reconstruction (Fig. 2).

The mean age was 53.7 (standard deviation 15.0) years, and the number of females was 23 (39.0%). Neoadjuvant chemoradiation therapy was performed more frequently in the ePTFE group with marginal significance (35.5% vs 14.3% in the ePTFE vs Bovine groups, *P *=* *0.08). In terms of primary disease, thymoma (*n* = 21, 35.6%) and thymic carcinoma (*n* = 17, 28.8%) were the 2 most frequent diagnoses, followed by germ cell tumour (*n* = 7, 11.9%), lung cancer (*n* = 3, 5.1%), sarcoma (*n* = 3, 5.1%) and thyroid cancer (*n* = 2, 3.3%) (Table 1).

**Table 1:** Preoperative characteristics and risk factors for the study patients

Variables	Total (*n* = 59)	ePTFE (*n* = 31)	Bovine (*n* = 28)	*P*-value
Age (years)	53.7 (SD 15.0)	55.3 (SD 13.4)	52.0 (SD 16.7)	0.41
Sex (female), *n* (%)	23 (39.0)	12 (38.7)	11 (39.3)	0.96
Body surface area (m^2^)	1.71 (SD 0.18)	1.71 (SD 0.19)	1.72 (SD 0.17)	0.78
Risk factors, *n* (%)				
Smoking	18 (30.5)	8 (25.8)	10 (35.7)	0.41
Body mass index > 25.0 kg/m^2^	17 (28.8)	8 (25.8)	9 (32.1)	0.59
Hypertension	12 (20.3)	7 (22.6)	5 (17.9)	0.75
Diabetes mellitus	5 (8.5)	3 (9.7)	2 (7.1)	>0.99
Dyslipidaemia	6 (10.2)	3 (9.7)	3 (10.7)	>0.99
History of stroke	2 (3.4)	2 (6.5)	0 (0.0)	0.49
Chronic kidney disease	1 (1.7)	1 (3.2)	0 (0.0)	>0.99
Chronic obstructive pulmonary disease	0 (0.0)	0 (0.0)	0 (0.0)	–
Atrial fibrillation	2 (3.4)	2 (6.5)	0 (0.0)	0.49
Redo-sternotomy	1 (1.7)	0 (0.0)	1 (3.6)	>0.99
Neoadjuvant treatment, *n* (%)				
Neoadjuvant chemotherapy	24 (40.7)	12 (38.7)	12 (42.9)	0.75
Neoadjuvant chemoradiation therapy	15 (25.4)	11 (35.5)	4 (14.3)	0.08
Primary disease, *n* (%)				
Malignant disease	57 (96.6)	29 (93.5)	28 (100.0)	0.49
Thymoma	21 (35.6)	12 (38.7)	9 (32.1)	
Thymic carcinoma	17 (28.8)	10 (32.3)	7 (25.0)	
Germ cell tumour	7 (11.9)	1 (3.2)	6 (21.4)	
Lung cancer	3 (5.1)	0 (0.0)	3 (10.7)	
Sarcoma	3 (5.1)	3 (9.7)	0 (0.0)	
Thyroid cancer	2 (3.3)	0 (0.0)	2 (7.1)	
Others	5 (8.5)	5 (16.1)	1 (3.6)	
Benign disease	2 (3.4)	2 (6.5)	0 (0.0)	0.49

Continuous variables are presented as the mean (SD) for normally distributed variables and as the median with interquartile range for non-normally distributed variables.

ePTFE: expanded polytetrafluoroethylene; SD: standard deviation.

### Operative data

The total number of grafts was 32 and 33 in the ePTFE and Bovine groups, respectively. In the ePTFE group, 16 mm grafts were most frequently used (*n* = 18, 58.1%), followed by 14 mm (*n* = 7, 22.6%), 18 mm (*n* = 4, 12.9%) and 12 mm (*n* = 2, 6.5%) grafts. In the Bovine group, the data regarding the size of the bovine pericardial conduit were limited because the sizing was dependent on the intraoperative measurements, and no objective values were recorded. Sacrifice of the other BCV (sacrifice of the left BCV in right BCV-SVC reconstruction or vice versa) was more frequently performed in the ePTFE group (81.3% vs 54.5% in ePTFE vs Bovine groups, *P *=* *0.02) (Table 2).

**Table 2:** Operative data of the study patients

Variables	ePTFE (*n* = 31)	Bovine (*n* = 28)	*P*-value
Total number of grafts, *n*	32	33	
Type of reconstruction, *n* (%)			
Right BCV	29 (93.5)	19 (67.9)	
Left BCV	1 (3.2)	4 (14.3)	
Bilateral BCV	1 (3.2)	5 (17.9)	
Graft size, *n* (%)			
12 mm	2 (6.5)	–	
14 mm	7 (22.6)	–	
16 mm	18 (58.1)	–	
18 mm	4 (12.9)	–	
Sacrifice of the other BCV, *n* (%)	26 (81.3)	18 (54.5)	0.02
Use of CPB, *n* (%)	5 (16.1)	3 (10.7)	0.30
Proximal anastomosis site, *n* (%)			0.26
SVC	30 (93.8)	27 (81.8)	
RAA	2 (6.3)	6 (18.2)	

BCV: brachiocephalic vein; CPB: cardiopulmonary bypass; ePTFE: expanded polytetrafluoroethylene; RAA: right atrial auricle; SVC: superior vena cava.

### Early and midterm clinical outcomes

Operative mortality rate was 1.7%. Postoperative complications are detailed in Supplementary Material, Table S1.

All-cause mortality occurred in 15 patients. Overall survival was 93.4% vs 87.9% at 1 year and 93.4% vs 78.8% at 2 years in the ePTFE and Bovine groups, respectively (*P *=* *0.17). SVC reintervention was performed in 4 patients. The cumulative incidence of SVC reintervention was 0.0% vs 3.0% at 1 year and 0.0% vs 9.1% at 2 years in the ePTFE and Bovine groups, respectively (*P *=* *0.41) (Supplementary Material, Fig. S1). Restricted mean survival times were also comparable between the groups (40.0 vs 37.5 months in the ePTFE and Bovine groups, *P *=* *0.08).

### Midterm graft patency

**Figure 3:**
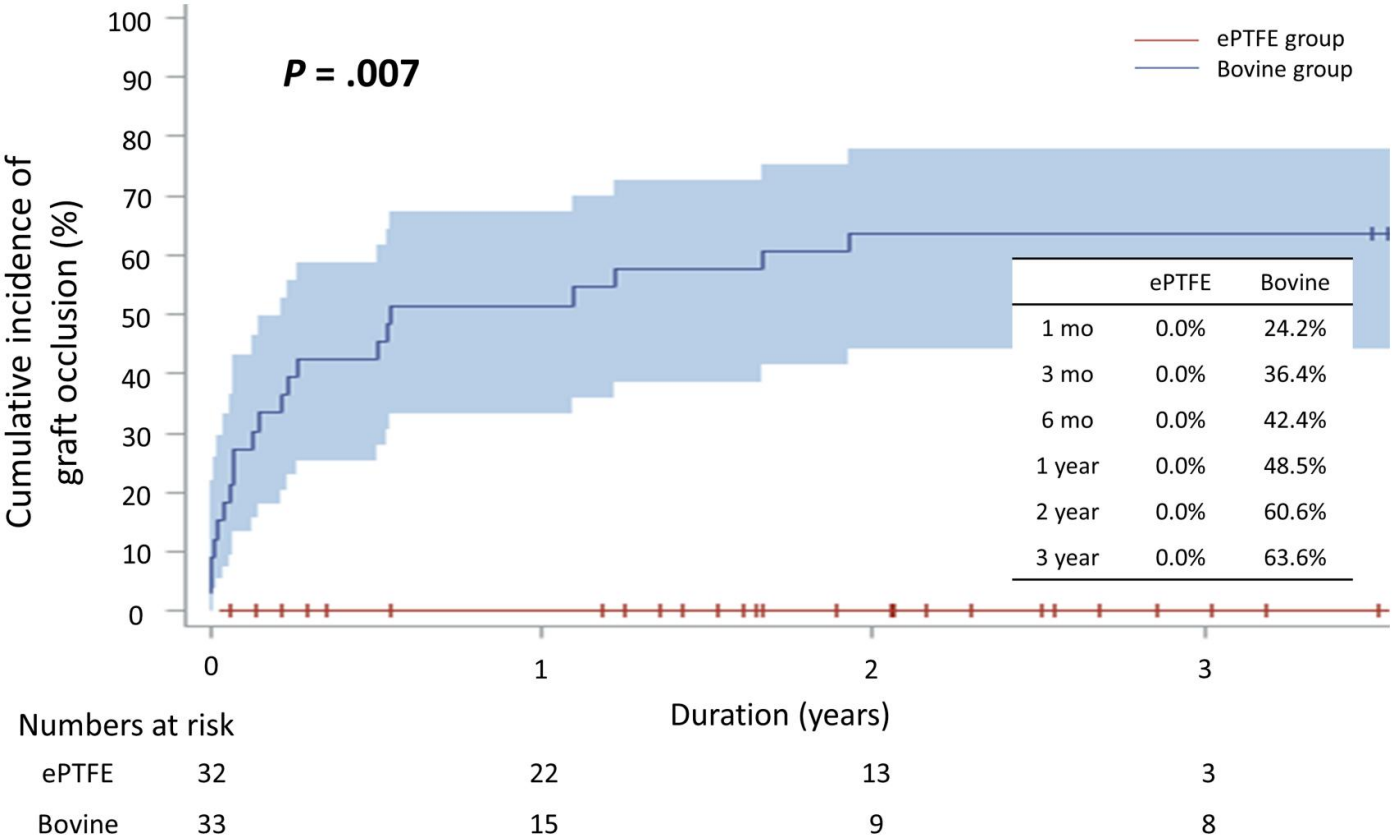
Comparison of midterm graft patency between the groups. ePTFE: expanded polytetrafluoroethylene.

Graft occlusion was detected in 22 grafts. The cumulative incidence of graft occlusion was 0.0% up to 2 years in the ePTFE group, whereas it was 24.2%, 36.4%, 42.4%, 48.5% and 60.6% at 1 month, 3 months, 6 months, 1 year and 2 years, respectively, in the Bovine group (*P *=* *0.007) (Fig. 3). In restricted mean survival time analyses, ePTFE group demonstrated significantly longer restricted mean survival time than Bovine group (40.0 vs 17.4 months, *P *<* *0.001).

### Risk factor analysis for graft occlusion

Univariate analysis demonstrated that left BCV reconstruction and bovine pericardial conduits were significant risk factors for midterm graft occlusion, while neoadjuvant chemoradiation therapy and sacrifice of the other BCV were significant protective factors. Multivariate analysis demonstrated that left BCV reconstruction was a significant risk factor (subdistribution hazard ratio 5.57, *P *<* *0.001) and ePTFE graft reconstruction was a protective factor (subdistribution hazard ratio 0.03, *P *=* *0.01) among the variables (Table 3).

**Table 3:** Multivariate analysis of the risk factors associated with midterm graft occlusion

	Univariate analysis			Multivariate analysis		
Variables	*P*-value	sHR	95% CI	*P*-value	sHR	95% CI
Sex (female)	0.32	1.52	0.67–3.45			
Age (years)	0.33	1.01	0.99–1.04			
Body surface area (m^2^)	0.52	0.47	0.05–4.56			
Smoking	0.63	0.79	0.31–2.03			
Body mass index >25.0 (kg/m^2^)	>0.99	1.00	0.39–2.58			
Hypertension	0.61	0.72	0.21–2.50			
Diabetes mellitus	0.63	0.58	0.06–5.31			
Dyslipidaemia	0.32	0.38	0.06–2.58			
History of stroke	0.48	0.35	0.00–2.55			
Chronic kidney disease	0.88	1.26	0.01–9.19			
Atrial fibrillation	0.72	0.59	0.01–4.28			
Redo-sternotomy	0.10	3.86	0.77–19.47			
Neoadjuvant chemotherapy	0.50	0.75	0.33–1.73			
Neoadjuvant chemoradiation therapy	0.08	0.25	0.06–1.15			
Malignant primary disease	0.86	1.29	0.18–164.6			
Type of reconstruction (left BCV reconstruction)^a^	<0.001	8.68	3.45–21.82	<0.001	5.57	2.26–13.31
Sacrifice of the other BCV	0.01	0.34	0.15–0.78			
Use of CPB	0.21	0.48	0.15–1.51			
Proximal anastomosis site^b^	0.006	3.80	1.47–9.82			
Type of prosthesis (ePTFE graft)^c^	0.007	0.02^d^	0–0.14	0.01	0.03^d^	0–0.18

a The reference for calculating the sHR was right BCV reconstruction.

b The reference for calculating the sHR was proximal anastomosis to SVC.

c The reference for calculating the sHR was a bovine pericardial conduit.

d Penalized maximum likelihood estimates.

BCV: brachiocephalic vein; CI: confidence interval; CPB: cardiopulmonary bypass; ePTFE: expanded polytetrafluoroethylene; sHR: subdistribution hazard ratio; SVC: superior vena cava.

## DISCUSSION

The present study demonstrated 3 main findings. First, the ePTFE graft demonstrated superior midterm patency compared with the bovine pericardial conduit. Second, right BCV reconstruction demonstrated superior midterm patency compared with left BCV reconstruction. Third, sacrificing the other BCV might have a positive effect on the midterm patency of the reconstructed graft.

**Figure 4:**
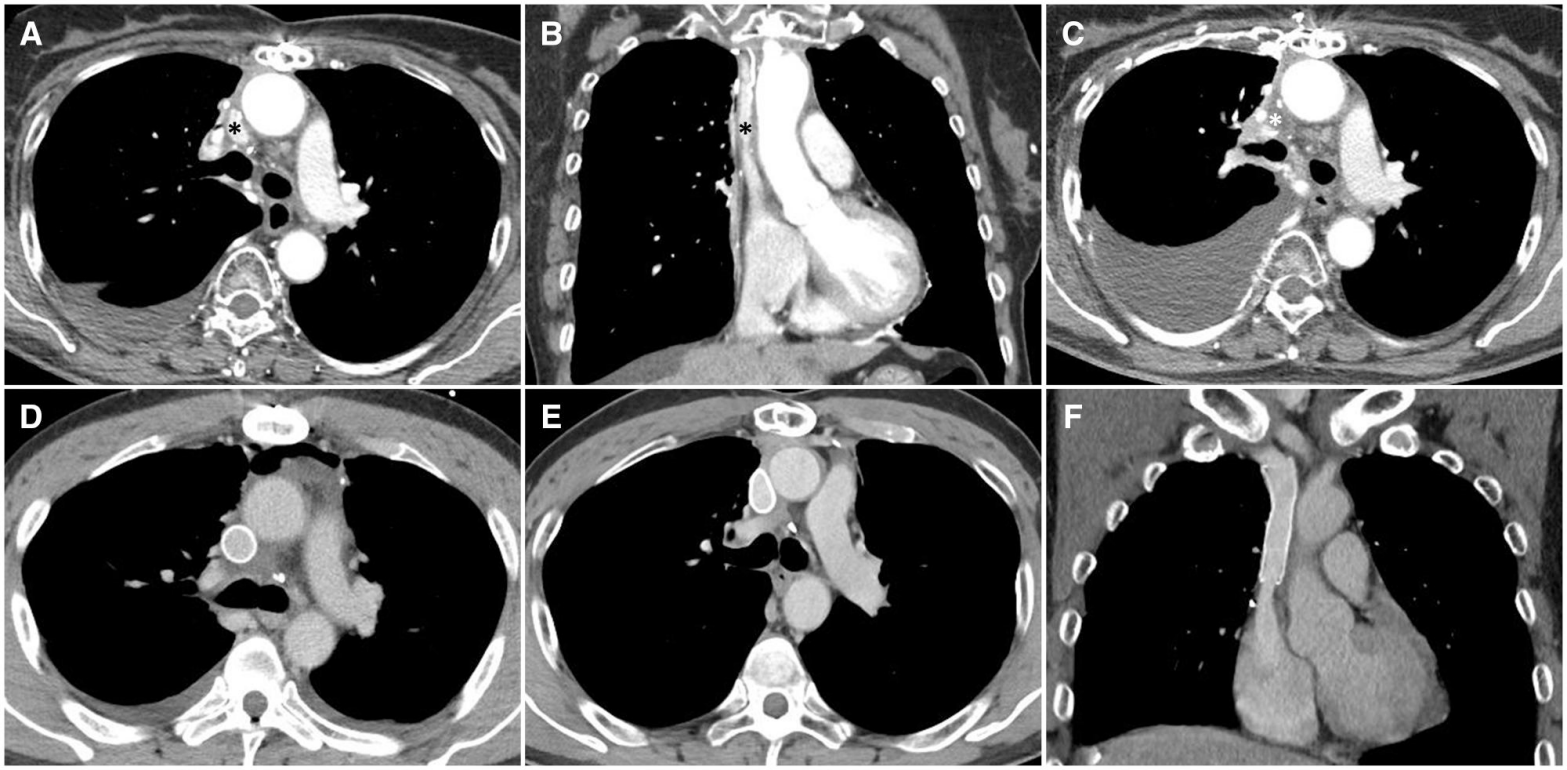
Serial CT scans of 2 patients who underwent SVC reconstruction with a bovine pericardial conduit and an ePTFE graft without rings. (**A** and **B**) The bovine pericardial conduit (black asterisk) was patent at 4 months after surgery but it showed a triangular shape compressed by surrounding structures, including the ascending aorta and expanded right lung. (**C**) Thrombotic occlusion of the bovine pericardial conduit (white asterisk) was confirmed 16 months after surgery. (**D**) The ePTFE graft without rings was patent at 5 days after surgery. (**E** and **F**) The patency of the ePTFE graft was confirmed at the 41-month follow-up CT scan. Despite external pressure from the surrounding structures and mediastinal fibrosis, the ePTFE graft exhibited an oval shape and maintained a sufficient luminal area due to its intrinsic rigidity. CT: computed tomography; ePTFE: expanded polytetrafluoroethylene; SVC: superior vena cava.

The advantages of a bovine pericardial conduit include its biocompatibility and reduced risk of infections and thrombosis [8, 10]. Furthermore, its thinner and more pliable nature makes it easier to suture and trim to the vascular wall [8]. However, there are also previous studies reporting suboptimal patency of bovine pericardial conduits [10, 11]. Unlike an ePTFE graft, a bovine pericardial conduit has an additional longitudinal suture line made by stapler, which might be thrombotic and result in graft occlusion. It also has no rigidity and is prone to external compression of surrounding structures, including the ascending aorta and expanded right lung. When compressed by these structures, the lumen of the bovine pericardial conduit changes to a sharp triangular shape or further slit-like shape and is prone to thrombotic occlusion. Mediastinal fibrosis and scar tissue formation can also affect the midterm patency of bovine pericardial conduits by distorting their luminal shape. The findings from the serial CT scans of a patient who presented with late occlusion of the bovine pericardial conduit support these theoretical mechanisms of occlusion in the bovine pericardial conduit (Fig. 4A–C).

Among the synthetic materials, the ringed ePTFE graft (Gore-Tex vascular graft; W.L. Gore & Associates, Inc., Flagstaff, AZ, USA) is used widespread and is regarded as the graft of choice based on previous studies [6, 12–14]. Reinforcing rings in ringed ePTFE grafts can better sustain external compression and can prevent graft collapse when the central venous pressure becomes negative [15]. Additionally, it has less platelet deposition and less thrombogenicity caused by re-epithelialization with autogenous cells [15].

However, due to the unavailability of the ringed ePTFE graft in Korea, we decided to use the ePTFE graft without rings, which shares the advantages of ringed ePTFE grafts, including less platelet deposition, less thrombogenicity and re-epithelialization. Its use was also based on the proven long-term outcome of the graft as an extracardiac conduit in Fontan patients [16]. Although it does not have reinforcing rings resistant to external pressure, it showed a sufficient luminal area in the follow-up CT scans in our series, which is attributed to its intrinsic rigidity (Fig. 4D–F).

Although some surgeons have advocated both BCVs reconstruction based on haemodynamic and oncologic reasons [13], the left BCV demonstrated inferior patency than the right BCV or the SVC due to the graft kinking, compression between sternum and aorta, slow flow velocity, and turbulence by RAA pectinate muscles [12, 17, 18]. In addition, unilateral BCV reconstruction with ligation of the other BCV is physiologically tolerated and showed satisfactory long-term patency [14, 15, 19]. Ligation of a BCV usually resulted in the increased blood flow passing through the contralateral BCV by the intracranial collaterals, and may contribute to the improved long-term patency of the reconstructed graft. No additional procedure, including an arterio-venous fistula formation in the ipsilateral arm, was required to increase the blood flow via graft. In our early experience, left BCV reconstruction using bovine pericardial conduit indeed showed dismal patency outcomes, and a right-side single-limbed graft showed satisfactory patency outcomes with tolerable haemodynamics and symptoms. These led us to abandon bilateral reconstruction and left BCV reconstruction strategies. In later series at our institution, we made it an institutional strategy to ligate the left BCV and perform graft reconstruction from the right BCV to the proximal SVC with extensive *en bloc* resection of the SVC if any extent of vessel wall involvement was suspected. This simple, straightforward and consistent strategy also helps to avoid any technical errors of kinking and to result in direct downwards blood flow aided by gravity [14]. It also enables extensive *en bloc* resection of the SVC, which provides a sufficient resection margin, improving the freedom from local recurrence.

In terms of intraoperative haemodynamic management, we routinely used a central venovenous shunt from distal right BCV to RAA to alleviate the potential risk of hypotension and to prevent congestion of the brain in the later period [9, 20–22]. In our series with partially or not obstructing the SVC, cerebral venous pressure increased to 30–40 mmHg immediately after total clamping of the SVC; however, cerebral venous pressure decreased to 15–20 mmHg after the initiation of the venovenous shunt.

There has been controversy regarding the role and the optimal duration of anticoagulation therapy after graft replacement of BCVs or SVCs. Although many authors have proposed long-term use of warfarin for 3–6 months [9, 13, 23, 24], some have questioned the need for anticoagulation therapy [14]. Despite the administration of antithrombotic agents, graft occlusion occurred in up to 38% of the patients, and most occlusions were observed within 1 month of reconstruction [9, 12, 23, 24], as in our study. A previous study demonstrated that graft occlusion was not associated with antithrombotic agent usage but largely associated with technical errors resulting in stenosis or kinking [14]. We only prescribed antiplatelet agents for long-term maintenance in ePTFE grafts, and no graft thrombosis or occlusion has been observed.

### Limitations

This study has several limitations that should be recognized. First, this was a single-center retrospective study with a small sample size and with a long study period. Second, the follow-up duration was relatively short to confirm the long-term patency of the grafts. Third, the 2 groups were biased by not uniform anticoagulation management, different surgeon factors and different perioperative management over time, which limited discriminating the efficacy of the prostheses on graft patency. Fourth, ethnic differences should be taken into consideration because Asians are known to have less thrombogenicity than Western people [25]. Fifth, the ringed PTFE grafts and autologous pericardial grafts, which were used only in 3 patients, respectively, were excluded from the analysis. At our institution, ringed PTFE grafts demonstrated no occlusion until 6 months, and autologous pericardial grafts demonstrated 1 occlusion at 6 months follow-up. Further investigation in large cohort is required to clarify the patency outcome between the grafts.

## CONCLUSION

For patients who underwent SVC resection and reconstruction, the PTFE graft was superior to the bovine pericardial graft in terms of midterm graft patency.

## Supplementary Material

ivae194_Supplementary_Data

## Data Availability

Data associated with the article are available on reasonable request to the corresponding author.

## Notes

Presented at the AATS 104th Annual Meeting, Toronto, Canada, 27 April 2024.

## ABBREVIATIONS

BCV: Brachiocephalic vein
CT: Computed tomography
ePTFE: Expanded polytetrafluoroethylene
RAA: Right atrial appendage
SVC: Superior vena cava
