# Supplementary material for: Prosthesis selection for reconstruction of superior vena cava: comparison of midterm patency rates
Source: Interdiscip Cardiovasc Thorac Surg. 2024 Nov 26;39(6):ivae194. doi: 10.1093/icvts/ivae194 (PMC11852341; doi:10.1093/icvts/ivae194)

**Supplementary material**

Supplementary Table 1. Comparison of early clinical outcomes between the groups.

| Variables | Total  (n = 59) | ePTFE  (n = 31) | Bovine  (n = 28) | *P* |
| --- | --- | --- | --- | --- |
| Operative mortality, n (%) | 1 (1.7) | 1 (3.2) | 0 (0.0) | >0.99 |
| Postoperative complications, n (%) |  |  |  |  |
| Postoperative atrial fibrillation | 6 (10.2) | 2 (6.5) | 4 (14.3) | 0.41 |
| Vocal cord palsy | 6 (10.2) | 2 (6.5) | 4 (14.3) | 0.41 |
| Chylothorax | 7 (11.9) | 5 (16.1) | 2 (7.1) | 0.43 |
| Pneumonia | 5 (8.5) | 3 (9.7) | 2 (7.1) | >0.99 |
| Tracheostomy | 1 (1.7) | 0 (0.0) | 1 (3.6) | >0.99 |
| Bleeding reoperation | 2 (3.4) | 1 (3.2) | 1 (3.6) | >0.99 |
| Delirium | 1 (1.7) | 1 (3.2) | 0 (0.0) | >0.99 |
| Hospital stay, days | 9 [7, 16] | 9 [7, 16] | 10 [8, 17] | 0.21 |

Continuous variables are presented as median with interquartile range for nonnormally distributed variables.

ePTFE, expanded polytetrafluoroethylene.

Supplementary Figure 1. Comparison of (A) overall survival and (B) the cumulative incidence of SVC reintervention between the groups.


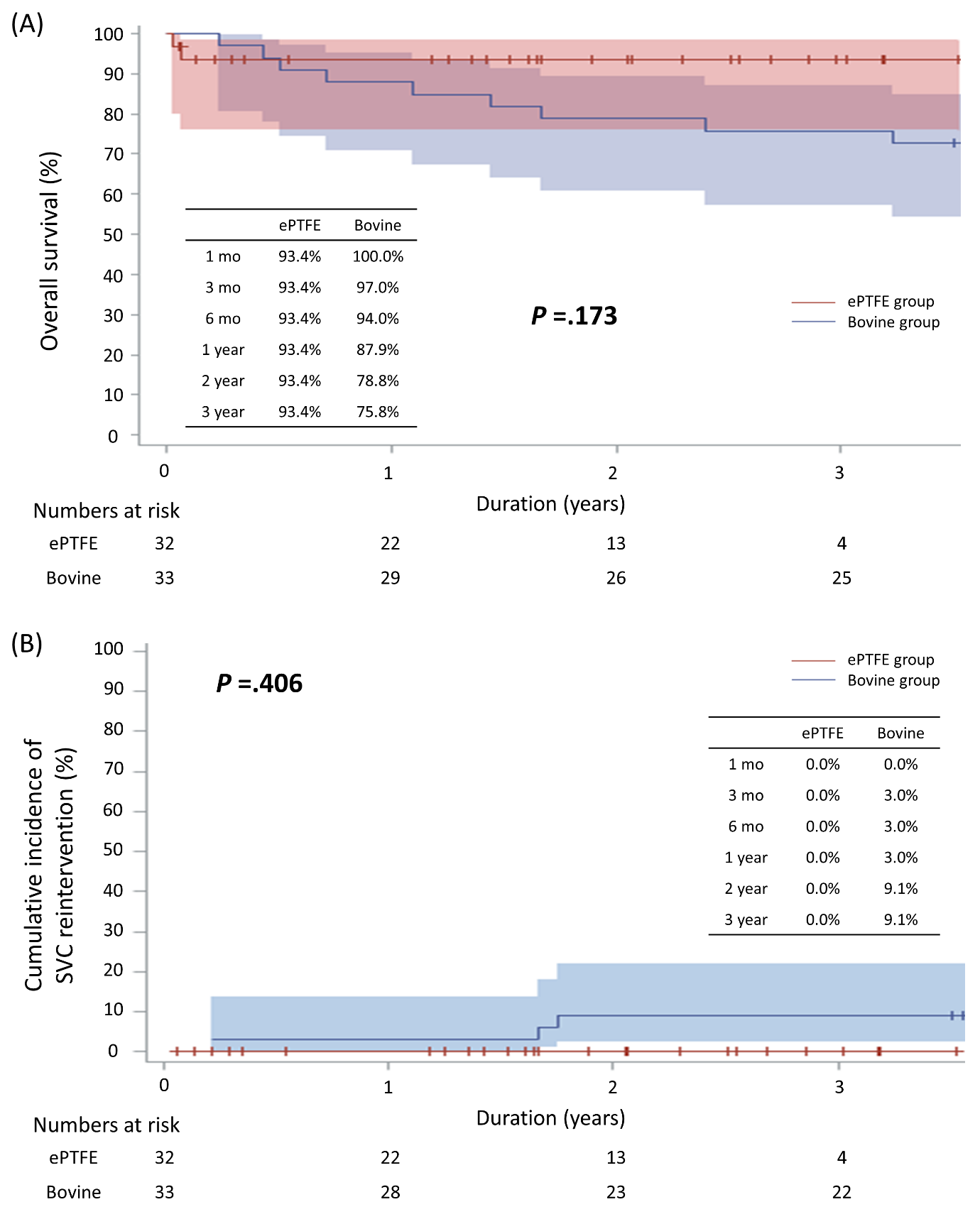

Supplement: ivae194_Supplementary_Data [file ivae194_supplementary_data.docx]
